# Predictors of the development of post-snakebite compartment syndrome

**DOI:** 10.1186/s13049-015-0179-y

**Published:** 2015-11-11

**Authors:** Chih-Po Hsu, Jung-Fang Chuang, Yu-Pao Hsu, Shang-Yu Wang, Chih-Yuan Fu, Kuo-Ching Yuan, Chih-Hao Chen, Shih-Ching Kang, Chien-Hung Liao

**Affiliations:** Department of Trauma and Emergency Surgery, Chang Gung Memorial Hospital, Chang Gung University, 5 Fu-Hsing Street, Kwei-Shan Shiang, Taoyuan Taiwan; Department of Trauma and Emergency Surgery, Kaohsiung Chang Gung Memorial Hospital and Chang Gung University College of Medicine, Kaohsiung, Taiwan; Division of Trauma Plastic Surgery, Chang Gung Memorial Hospital, Chang Gung University, Taoyuan, Taiwan

## Abstract

**Background:**

To identify the factors associated with the development of post-snakebite compartment syndrome (PSCS) in snakebite patients and to analyze the clinical prognosis of these patients.

**Methods:**

We retrospectively reviewed the medical records of patients who presented to our institution with snakebites from March 2009 to December 2012. The clinical data, hospital course and outcome were all recorded.

**Results:**

A total of 136 patients were included in the present study. Nine patients developed PSCS and underwent fasciotomy. Relative to the non-PSCS group, the PSCS group demonstrated a significant increase in the white blood cell count (WBC, *p =* 0.006), segment form (Seg, *p ≤* 0.001), aspartate aminotransferase level (AST, *p =* 0.002) and alanine aminotransferase level (ALT, *p =* 0.008). Elevated WBC count and AST level were identified as independent risk factors for PSCS (*p =* 0.028 and 0.037, respectively) in a multivariate analysis.

**Conclusions:**

Snakebite patients have a high likelihood of developing locoregional complications such as PSCS. Symptomatic snakebite patients should be observed for at least 48 h, and increased WBC counts and AST levels are risk factors for PSCS.

## Introduction

Snakebites remain a public health problem worldwide. The clinical presentations of snakebites vary from symptomless to life threatening [1]. Snakebites require vigilance regarding both the systemic effects of the venom and the locoregional impact on the soft tissues. The widespread use of antivenom has allowed the systemic effect of venom to be controlled with an acceptable prognosis. Locoregional tissue injury commonly results in pain, blistering, and bruising. Severe tissue necrosis induces the development of compartment syndrome, which is a rare but lethal complication after a snakebite. Nevertheless, there have been limited reports concerning post-snakebite compartment syndrome (PSCS) [2, 3], and there is a lack of consensus regarding the diagnosis and management of PSCS.

When compartment syndrome has been diagnosed, fasciotomy should be performed immediately to prevent permanent damage to the muscles and nerves of the affected limbs [4, 5]. However, the development of PSCS after envenomation may span several hours or even days. Therefore, close monitoring remains the method of choice for detecting PSCS. Because of a lack of adequate predictors of PSCS in the available literature, physicians face a dilemma in the management of snakebite patients, as prolonged monitoring is associated with increased medical costs.

In this study, we present our experience with the management of snakebites at a trauma center. We identified factors associated with the development of PSCS in snakebite patients and analyzed the prognosis of these patients.

## Materials and methods

### Data collection

We prospectively collected data from the trauma registry at Chang Gung Memorial Hospital (CGMH), Linkou, which is a level I trauma center in Taiwan. We prospectively recorded the following information from a computerized trauma registration system database: demographic data; trauma mechanism; prehospital, medical, perioperative, and hospital course; follow-up information; and information regarding complications. We retrospectively reviewed the records of patients with snakebites who were managed at our institution from March 2009 to December 2012. The cases, including the snakebite diagnoses, were filtered utilizing International Classification of Diseases, Ninth Revision, Clinical Modification (ICD-9-CM) codes (E905.0). The dataset was further limited by age to include patients over 18 years old and younger than 80 years old. The demographic and diagnostic data abstracted from the medical records included the following: age, gender, time from injury to emergency department (ED) admission, snake species, clinical parameters at presentation, laboratory data, antivenom dosage, development of PSCS, hospitalization requirement, length of hospital stay and complications. The Institutional Review Board of Chang Gung Memorial Hospital approved the study.

### Treatment protocol

**Fig. 1:**
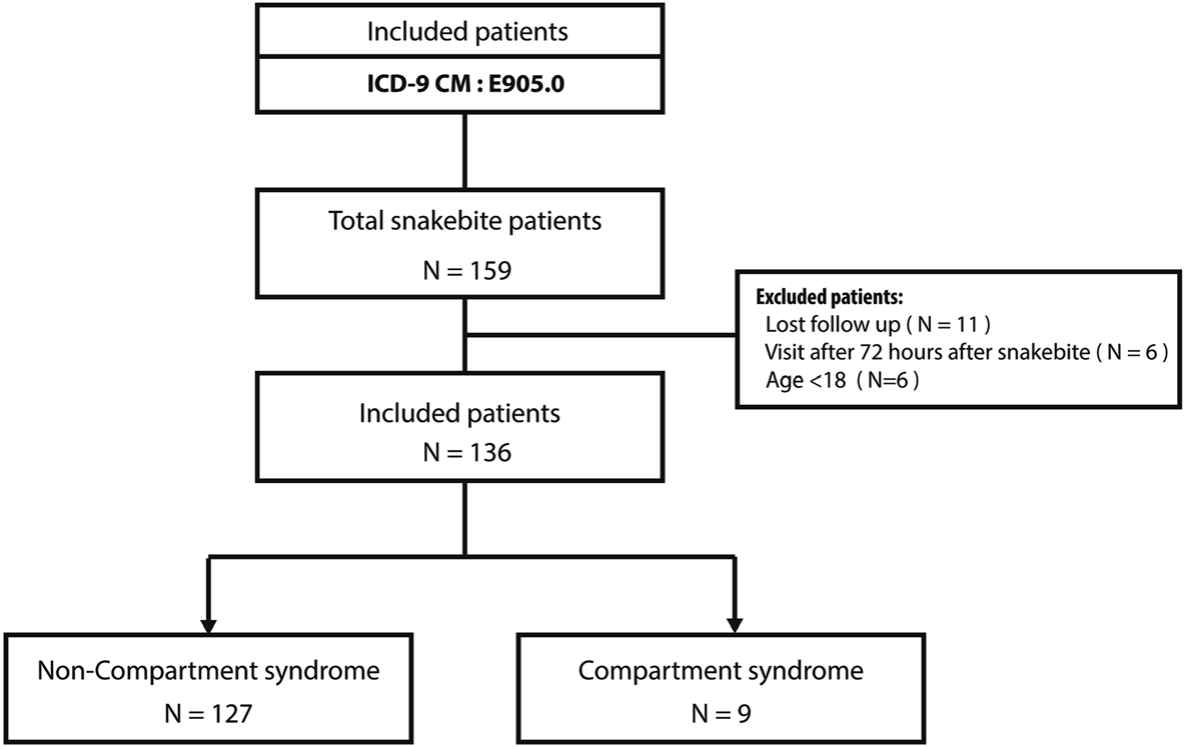
Consort diagram describing cohort identification. ICD-9-CM: International Classification of Diseases, Ninth Revision, Clinical Modification

The treatment protocol for snakebites at our institution has been used for years. The first step is to identify the species responsible for the bite. Six common venomous snakes are found in Taiwan: the Taiwan Habu (*Trimeresurus mucrosquamatus*), bamboo viper (*Trimeresurus stejnegeri*), Russell’s pit viper (*Daboia russellii formosensis*), Taiwan cobra (*Naja naja atra*), Taiwan banded krait (*Bungarus multicinctus*) and sharp-nosed pit viper (*Deinagkistrodon acutus*) [6, 7]. The snake species involved is confirmed primarily by the patient, family or medical staff. If the snake species cannot be identified, the snake is categorized as an unknown species. Asymptomatic patients who do not show local symptoms after 2 h are typically discharged. However, symptomatic patients are monitored for at least 24 h. All symptomatic patients receive adequate antivenom intravenously with a recommended dosage according to the species. For unknown species, we apply both hemorrhagic and neurotoxic antivenom and monitor the response. Additional doses are given if the clinical presentation does not improve. For patients with progressive symptoms, hospitalization is arranged. Other treatments, including antihistamines, corticosteroids, and antibiotic agents, are used according to clinical judgment. The 6 “P”s are used to diagnose PSCS: pain, paresthesia, pallor, paralysis, poikilothermia and pulselessness. If the patients present one of the 6 “P”s, we consider that to indicate impending compartment syndrome. If patients present with more than two “P”s, compartment syndrome is clinically diagnosed. Fasciotomy is indicated if PSCS is diagnosed in the distal limbs or if the intracompartmental pressure approaches 20 mmHg below the diastolic pressure. In the present study, we excluded patients who visited the ED more than 72 h after the snakebite episode or who were lost to follow-up at our institution (Fig. 1).

### Statistical analysis

Pearson’s *χ*^2^ test and Fisher’s exact test were used to compare categorical variables. Quantitative variables were compared using Student’s *t*-test and the Mann–Whitney *U* test. Levene’s test was used to correct for intergroup variations before conducting Student’s *t*-test. All statistically significant factors in the univariate analysis were included in further multivariate analysis by logistic regression. Receiver operating characteristic (ROC) curves were used to identify the sensitivity and specificity, and Youden’s index was used to identify the best cut-off values for the diagnosis of compartment syndrome. A value of *p* < 0.05 was considered to be statistically significant. All statistical analyses were performed using SPSS v17.0 (SPSS Inc., Chicago, IL, USA).

## Results

Out of 159 patients with snakebites, 136 patients were enrolled in this study. The mean age of the enrolled patients was 51.5 ± 17.0 years. There were 101 males (74.3 %) and 35 females (25.7 %). There were 84 patients (61.7 %) with locoregional symptoms and 62 patients (45.6 %) with infection that required hospitalization. The patient demographics and outcomes are outlined in Table 1*.*

**Table 1:** Demographic characteristics of patients with and without post-snakebite compartment syndrome

Characteristics	All patient (N = 136)	Non-PSCS group (N = 127)	PSCS group (N = 9)	*p* value*
Age (years) (mean ± SD)	51.5 ± 17.0	51.4 ± 17.0	52.2 ± 17.2	0.955
Gender (n, %)				
Male	101 (74.3 %)	95 (74.8 %)	6 (66.7 %)	0.694
Female	35 (25.7 %)	32 (25.2 %)	3 (33.3 %)	
Time from injury to ED (hours)	5.8 ± 11.2	5.8 ± 11.5	5.8 ± 6.2	0.069
Snake species (n, %)				
Unknown species	72 (52.9 %)	67 (52.8 %)	5 (55.6 %)	0.996
Taiwan Habu (*Trimeresurus mucrosquamatus*)	31 (22.8 %)	29 (22.8 %)	2 (22.2 %)	
Bamboo viper (*Trimeresurus stejnegeri*)	17 (12.6 %)	16 (12.6 %)	1 (11.1 %)	
Russell’s pit viper (*Daboia russellii formosensis*)	3 (2.2 %)	3 (2.4 %)	0 (0.0 %)	
Taiwan cobra (*Naja naja atra*)	12 (8.8 %)	11 (8.7 %)	1 (11.1 %)	
Taiwan banded krait (*Bungarus multicinctus*)	1 (0.7 %)	1 (0.8 %)	0 (0 %)	
Initial GCS (n, %)				
GCS = 15	136 (100 %)	127 (100 %)	9 (100 %)	1.000
Initial blood pressure (n, %)				
SBP ≥ 90 mmHg	136 (100 %)	127 (100 %)	9 (100 %)	1.000
Laboratory data				
WBC (/uL)(mean ± SD)	8910 ± 3710	8600 ± 3500	12660 ± 4360	**0.006**
Seg (%) (mean ± SD)	64.4 ± 14.7	62.9 ± 14.1	82.7 ± 6.8	**<0.001**
INR (mean ± SD)	1.03 ± 0.92	1.03 ± 0.07	1.13 ± 0.24	0.155
Cr (mg/dL) (mean ± SD)	0.87 ± 0.22	0.86 ± 0.22	0.88 ± 0.19	0.525
AST (U/L) (mean ± SD)	37.9 ± 29.5	30.96 ± 12.68	103.00 ± 64.05	**<0.001**
ALT (U/L) (mean ± SD)	26.1 ± 15.3	24.82 ± 14.64	40.43 ± 16.65	**0.008**
Myoglobin (ng/mL) (median, IQR)	34.7 (20.8 – 64.3)	33.1 (20.0 – 59.7)	52.0 (34.3 – 1259.4)	0.123
CRP (mg/L) (median, IQR)	1.0 (0.4 -4.9)	1.0 (0.4 -4.9)	2.8 (0.9 -6.4)	0.275
Admission (n, %)				
Yes	62 (45.6 %)	53 (41.7 %)	9 (100 %)	**0.001**
No	74 (54.4 %)	74 (58.3 %)	0 (0 %)	
Length of hospital stay (day) (mean ± SD)	4.2 ± 6.7	3.3 ± 4.2	17.2 ± 16.7	**<0.001**

*The comparison is between non-PSCS and PSCS groups

*PSCS* Post-snakebite Compartment syndrome, *SD* standard deviation. *IQR* Interquartile range, *ED* emergency department, *GCS* Glasgow Coma Scale, *SBP* systolic blood pressure, *WBC* white blood count, *Seg* segment, *PLT* platelet, *INR* international normalized ratio, *APTT* activated partial thromboplastin time, *BUN* blood urea nitrogen, *Cr* creatinine, *AST* aspartate aminotransferase, *ALT* alanine aminotransferase, *CRP* C reactive protein

Bold data represent the *p* value < 0.05

In the present study, the incidence of PSCS was 6.6 % (*n* = 9). We divided patients into two groups: the PSCS group (9 patients) and the non-PSCS group (127 patients). All clinical and laboratory factors were compared between these groups; the comparisons are summarized in Table 1. Compared to the non-PSCS group, the PSCS group displayed significantly higher WBC (*p* = 0.006), Seg (*p* < 0.001), AST (*p* = 0.002) and ALT (*p* = 0.008). A multivariate analysis showed that abnormal WBC counts and AST levels were independent factors (*p* = 0.028 and *p* = 0.037, respectively) for predicting the development of PSCS (Table 2). ROC curve analysis was performed for WBC and AST. The areas under curve for WBC and AST were 0.775 and 0.852, yielding cutoff values of 11,650/μL and 33.5 U/L, respectively. The sensitivity and specificity of a WBC count greater than 11,650/μL were 66.7 % and 83.6 %, respectively. The sensitivity and specificity of an AST level greater than 33.5 U/L were 85.7 % and 78.9 %, respectively.

**Table 2:** Multivariate analysis of factors that are predictive of compartment syndrome

	*p* value*	Cutoff value	Sensitivity	Specificity	Odds ratio	CI
WBC	0.028	11650/μL	66.7 %	83.6 %	10.53	2.42 - 45.79
Seg	NS	-	-	-	-	-
AST	0.037	33.5 U/L	85.7 %	78.9 %	14.25	1.63 - 124.81
ALT	NS	-	-	-		

*Abbreviations*: *WBC* white blood cell count, *Seg* segment, *AST* aspartate aminotransferase, *ALT* alanine aminotransferase, *NS* non-significant. *Forward conditional method

The clinical presentation and prognosis of the nine patients with PSCS are summarized in Table 3. All patients visited our ED within 24 h after the snakebite. The range of time from the ED to the OR was 1.40 to 39.73 h. Six of our patients suffered from established ischemia and tissue necrosis, and the other three had impending ischemic changes. All of the patients received multiple operations for wound closure after fasciotomy. One limb could not be preserved, and the amputation was performed. There was no mortality in any of the included snakebite cases.

**Table 3:** Summary of patients with post-snakebite compartment syndrome

Case	Age / Gender	WBC (/uL)	AST (U/L)	LOS (day)	Time from injury to ED (hours)	Time from ED to OR (hours)	Injury site	Presence of ischemia/ necrosis	Procedure times	1^st^ procedure	2^nd^ procedure	3^rd^ procedure
1	56/M	5900	239	37.79	6.2	2.46	Forearm	-	2	Fasciotomy	Wound repair	
2	63/M	11700	118	12.78	2.23	1.40	Forearm	+	3	Fasciotomy	Debridement advance flap	Debridement
3	53/M	16500	34	5.44	0.05	2.82	Foot	+	2	Fasciotomy	Advance flap	
4	55/M	15000	82	52.86	3.48	39.73	Hand	+	2	Fasciotomy	S.T.S.G	
5	75/F	15500	27	8.19	4.27	9.05	Leg	+	3	Left B-K amputation	Revision of B-K amputation debridement	
6	21/F	19400	109	6.59	4.93	3.91	Foot	+	2	Fasciotomy	Debridement advance flap	
7	77/F	9200	93	4.81	3.43	2.31	Hand	-	2	Fasciotomy	Debridement	
8	54/M	12500	150	18.02	21.45	28.16	Hand	+	3	Fasciotomy	Debridement wound repair	Wound revision
9	56/M	8200	75	8.62	6.77	2.61	Hand	-	2	Fasciotomy	Wound repair	

*ED* emergency department, *OR* operation room, *WBC* white blood count, *AST* aspartate aminotransferase, *LOS* Length of hospital stay, *S.T.S.G* splint-thickness skin graft, *B-K* below-knee

## Discussion

Since the development of the venom vaccine, snakebite-associated mortality has decreased [8, 9]. There was no mortality in the snakebite patients in this series. Therefore, the management of morbidity after snakebites is the most important consideration. Compartment syndrome is characterized by an increase in intracompartmental pressure with subsequent neurovascular compromise and tissue necrosis. The clinical signs and symptoms of compartment syndrome include pain, paresthesia, pallor, paralysis, poikilothermia and pulselessness [4, 10]. Several hypotheses have been proposed to explain the development of PSCS. Snake venom is composed of a chemically complex mixture that forms enzymatically active proteins after being injected into the human body. After the deposition of venom into subcutaneous tissue, a poison-induced capillary leak may induce extravasation of plasma and erythrocytes, resulting in edema and ecchymosis [11]. Animal studies have shown that venom deposited intramuscularly causes the release of tissue fluids into the compartment, resulting in elevated pressure [12]. The development of compartment syndrome is potentially lethal and may require limb amputation. The incidence of PSCS was rare in previous reports (0.20-1.36 %) [2, 3, 10]; however, the incidence of PSCS was 6.6 % in the current study. This higher incidence may be related to the predominance of cytotoxic snakes in Taiwan [7]. Three venomous snakes in Taiwan produce hemotoxins, including the sharp-nosed pit viper, Taiwan Habu and bamboo viper. A mix of hemotoxins and neurotoxins is secreted by Russell’s pit viper. The Taiwan cobra produces neurotoxins, but the venom is cytotoxic, causing local effects. Thus, the large number of snakebites in Taiwan involving cytotoxic venom may explain the high incidence of tissue necrosis leading to compartment syndrome.

The identification of the snake species responsible for a snakebite is crucial for optimal management [9]. Different snake species produce specific venoms that affect further treatments. Envenomation may be related to several factors involving the victim and the snake. Even the same species of snake may induce different injuries in different victims. Factors such as the size of the victim’s limb, size of the snake, efficiency of the bite, and contents of the venom apparatus at the time of the bite can affect the severity of the snakebite [3, 13]. Therefore, the snake species alone is not the sole factor that can be used to predict PSCS. In daily practice, it is very difficult for the patient or family to identify the species of snake, which can be problematic for the physician. However, the risk of further injury massively outweighs the benefit of chasing the animal for the purpose of identification.

After recognizing that knowledge of the snake species is not very useful for predicting PSCS, we assessed other risk factors that could predict PSCS and therefore aid clinical physicians in the management of snakebite patients. Close monitoring and compartment pressure measurement are standard approaches in the diagnosis of compartment syndrome. However, not all snakebite patients show immediate signs of compartment syndrome. Snakebite patients, especially asymptomatic patients, cannot all remain in the hospital to monitor progression. Furthermore, measuring intracompartmental pressure in all snakebite patients for the early detection of acute compartment syndrome is impractical. The ischemic signs of compartment syndrome, excluding severe pain, are late signs and should not be relied on for the early diagnosis of compartment syndrome. Therefore, identifying risk factors for compartment syndrome when patients arrive at the ED has become important. In this study, we found that elevated WBC (*p* = 0.028) and AST (*p* = 0.037) were risk factors for the development of PSCS, and further surgery was required. In addition, the cutoff values for WBC and AST were 11,650/μL and 33.5 U/L, respectively. These values may indicate an increase in the inflammatory or cytokine reaction after a severe snakebite and envenomation. Proteins and cytokines produced during the acute phase will induce further leukocytosis [14]. In addition, increased AST can be found in skeletal muscle and red blood cells. Acute hemolysis and necrosis of skeletal muscle in severe envenomation may release AST into the circulation [15]. These factors may explain why WBC and AST are useful predictors of PSCS. In symptomatic snakebite patients who have elevated WBC or AST, clinicians should anticipate the development of compartment syndrome.

Patients with asymptomatic snakebites are often discharged after being observed for two hours. However, the necessary duration of observation for symptomatic snakebite patients is controversial. In some studies, the authors suggested that symptomatic cases should be monitored for a minimum of 24 h [1, 10]. In the present study, all patients with compartment syndrome developed the condition between 1.4 and 39.7 h after ED arrival. Additionally, 22.2 % of patients (2/9) developed compartment syndrome after more than 24 h. In our department, to exclude the possibility of compartment syndrome, snakebite patients are carefully observed for local effects at least 48 h after admission.

Although the results of this study offer valuable insights into snakebites, the study has some limitations. First, this was a retrospective review at a single trauma center. Most characteristics were recorded prospectively, but the possibility of selection bias cannot be excluded. Second, although snakebites in Taiwan remain a public health problem, the number of victims has decreased due to rapid urbanization and snake habitat degradation. The small number of cases in this series may lead to statistical errors. Although our results indicate that increased WBC and AST are risk factors for the development of PSCS, our evidence may be insufficient. Therefore, it may not be safe to discharge patients based only on these markers. However, our results may be useful in the development of a decision tool to identify high- and low-risk patients in the future. We believe that increases in these markers combined with clinical presentation could be early diagnostic tools and aim to conduct a prospective study to validate these markers.

## Conclusions

PSCS is a critical problem that requires multiple surgical interventions. Elevated WBC and AST upon ED arrival are highly likely to be risk factors for the development of PSCS and may be useful as clinical markers. Thus, patients with snakebites and locoregional symptoms with elevated markers should be observed for 48 h to exclude the possibility of PSCS. In the future, there may be an opportunity to develop a decision tool that combines observations of clinical symptoms and measurement of WBC and AST levels. Such a tool may be a reasonable and safe way to distinguish patients who can be discharged without needing the 48-h observation period from those who may require surgery.

## Abbreviations

PSCS: Post-snake bite compartment syndrome
WBC: White blood count
AST: Aspartate aminotransferase
ALT: Alanine aminotransferase
ED: Emergency department
ICD-9-CM: International Classification of Diseases, Ninth Revision, Clinical Modification

## References

[1] Alkaabi JM, Neyadi Al M, Darei Al F, Mazrooei Al M, Yazedi Al J, Abdulle AM (2011). Terrestrial snakebites in the South East of the Arabian Peninsula: patient characteristics, clinical presentations, and management. PLoS One.

[2] Juckett G, Hancox JG (2002). Venomous snakebites in the United States: management review and update. Am Fam Physician.

[3] Frangides CY, Koulouras V, Kouni SN, Tzortzatos GV, Nikolaou A, Pneumaticos J (2006). Snake venom poisoning in Greece. Experiences with 147 cases. Eur J Intern Med.

[4] Köstler W, Strohm PC, Südkamp NP (2004). Acute compartment syndrome of the limb. Injury.

[5] Evers LH, Bartscher T, Lange T, Mailänder P (2010). Adder bite: an uncommon cause of compartment syndrome in northern hemisphere. Scandinavian Journal of Trauma, Resuscitation and Emergency Medicine.

[6] Chen Y-C, Chen M-H, Wang L-M, Wu JJ-K, Huang C-I, Lee C-H (2007). Antivenom therapy for crotaline snakebites: has the poison control center provided effective guidelines?. J Formos Med Assoc.

[7] Hung D-Z (2004). Taiwan's venomous snakebite: epidemiological, evolution and geographic differences. Trans R Soc Trop Med Hyg.

[8] Haidar NA, Deitch E (2015). Snake bites in the Arabian Peninsula, a review article. Journal of Arid Environments.

[9] Alirol E, Sharma SK, Bawaskar HS, Kuch U, Chappuis F (2010). Snake bite in South Asia: a review. PLoS Negl Trop Dis.

[10] Hall EL (2001). Role of surgical intervention in the management of crotaline snake envenomation. Ann Emerg Med.

[11] Gold BS, Barish RA, Dart RC, Silverman RP, Bochicchio GV (2003). Resolution of compartment syndrome after rattlesnake envenomation utilizing non-invasive measures. J Emerg Med.

[12] Garfin SR, Castilonia RR, Mubarak SJ, Hargens AR, Russell FE, Akeson WH (1984). Rattlesnake bites and surgical decompression: results using a laboratory model. Toxicon.

[13] Russell FE (1980). Snake venom poisoning in the United States. Annu Rev Med.

[14] Petricevich VL (2004). Cytokine and nitric oxide production following severe envenomation. Curr Drug Targets Inflamm Allergy.

[15] Bhagwat K, Amar L (2013). Blood hemoglobin, lactate dehydrogenase and total creatine kinase combinely as markers of hemolysis and rhabdomyolysis associated with snake bite. IJTPR.

